# The immune landscape of systemic sclerosis: from pathogenic mechanisms to precision therapeutic breakthroughs

**DOI:** 10.3389/fimmu.2026.1713221

**Published:** 2026-03-02

**Authors:** Mengguo Liu

**Affiliations:** Department of Dermatology, Huashan Hospital, Fudan University, Shanghai, China

**Keywords:** cellular crosstalk, fibrosis, immune microenvironment, precision medicine, systemic sclerosis, targeted therapy, vasculopathy

## Abstract

Systemic sclerosis (SSc) is a chronic autoimmune disease characterized by immune dysregulation, microvascular damage, and multi-organ fibrosis. Recent breakthroughs in single-cell and spatial multi-omics technologies have profoundly revealed the high heterogeneity of the SSc immune microenvironment, including extensive aberrant activation of innate immunity (e.g., dendritic cells, macrophages, neutrophils) and adaptive immunity (T cells, B cells), and their interaction with fibroblasts and endothelial cells through an “immune-stromal-vascular” network that collectively drives the fibrotic process. These findings have advanced disease subtyping based on molecular features (e.g., inflammatory, fibrotic) and the development of precision therapeutic strategies. Emerging therapies targeting the IL-6 receptor (tocilizumab), B cells (rituximab, belimumab, CAR-T), the JAK-STAT pathway (tofacitinib, baricitinib), and T-cell co-stimulation (abatacept) have shown potential to improve disease progression in clinical studies. However, heterogeneity in treatment response, difficulty in reversing fibrosis, and the lack of biomarkers remain current challenges. Future efforts require integrating multi-omics and artificial intelligence technologies to build dynamic predictive models, promoting multi-target combination and individualized therapies, ultimately aiming for early intervention and long-term remission in SSc.

## 1 Introduction

Systemic sclerosis (SSc) is a debilitating chronic autoimmune disease defined by three interconnected pathological hallmarks: progressive fibrosis of the skin and internal organs, microvascular dysfunction, and pervasive immune system dysregulation. Its clinical heterogeneity is a defining challenge—manifestations range from limited skin involvement to life-threatening organ damage affecting the lungs, heart, kidneys, and gastrointestinal tract, culminating in substantial morbidity and mortality. While the exact etiology of SSc remains elusive, decades of research have established that its pathogenesis arises from an intricate interplay of genetic predisposition (e.g., variants in HLA-DRB1, STAT4), environmental triggers (silica dust, organic solvents, viral infections), and aberrant activation of both innate and adaptive immune axes.

In recent years, the advent of high-throughput omics technologies has revolutionized our understanding of SSc by enabling systematic mapping of its immune landscape at single-cell and spatial resolutions. These tools have uncovered previously unrecognized immune cell subset heterogeneity, disrupted intercellular communication networks, and key pathogenic pathways that evaded traditional bulk analysis methods. This paradigm shift has not only deepened our grasp of SSc immunopathology but also opened unprecedented avenues for developing precision therapeutic strategies targeting disease-driving immune nodes.

**Figure 1:**
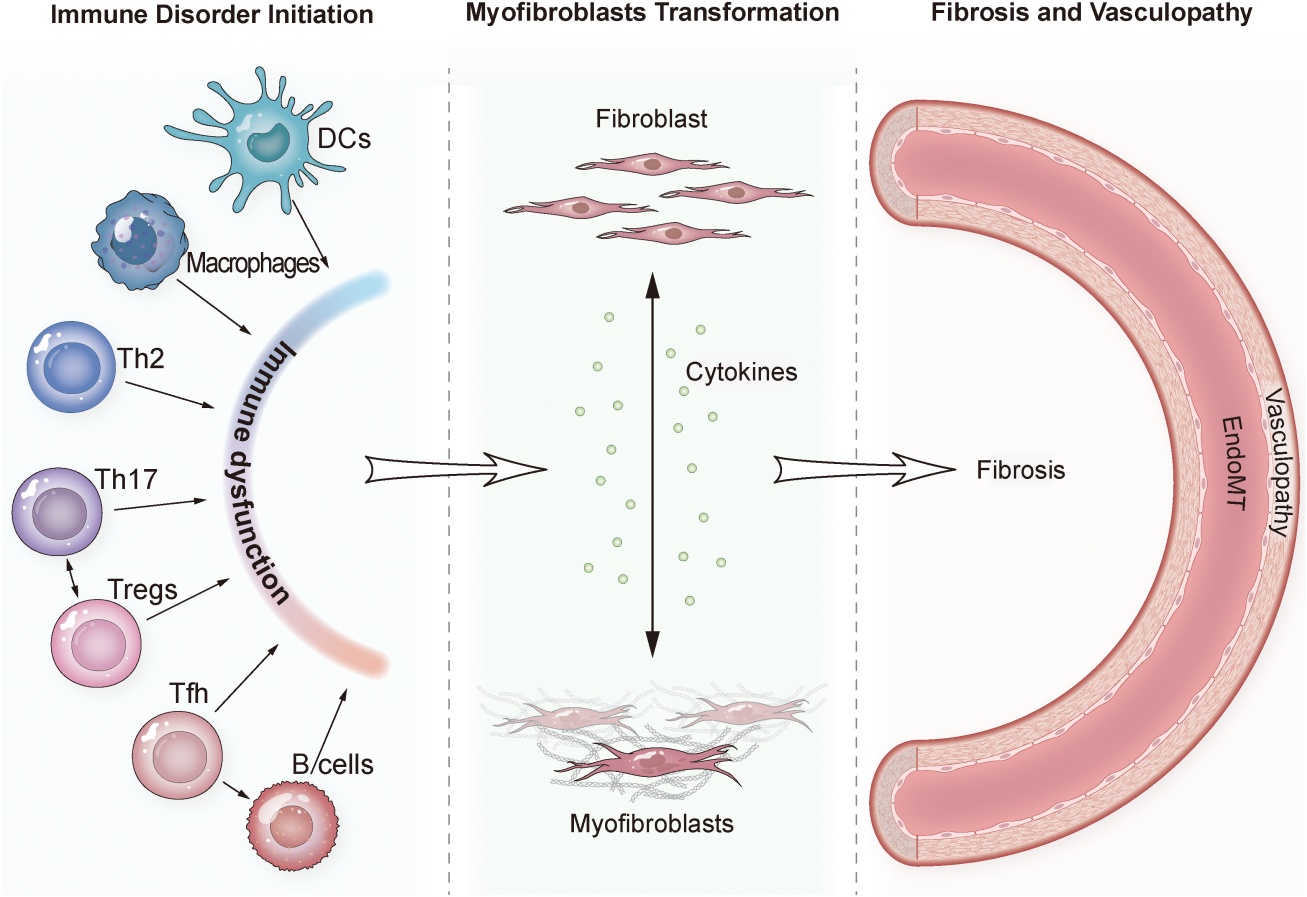
Schematic diagram of the "Immune-Stromal-Vascular" triad in Systemic Sclerosis pathogenesis. The illustration shows the aberrant crosstalk between immune cells (innate and adaptive), fibroblasts, and endothelial cells. Key interactions include: (1) Immune disorder initiation (involving DCs, macrophages, Th2, Th17, Tregs, Tfh, and B cells); (2) Myofibroblasts transformation (cytokines promote the transformation of fibroblasts into myofibroblasts); (3) Fibrosis and vasculopathy (involving Endothelial-to-Mesenchymal Transition, EndoMT). DC, Dendritic Cell; EndoMT, Endothelial-to-Mesenchymal Transition; Tfh, Follicular Helper T cell; Th, T helper cell; Treg, Regulatory T cell.

**Figure 2:**
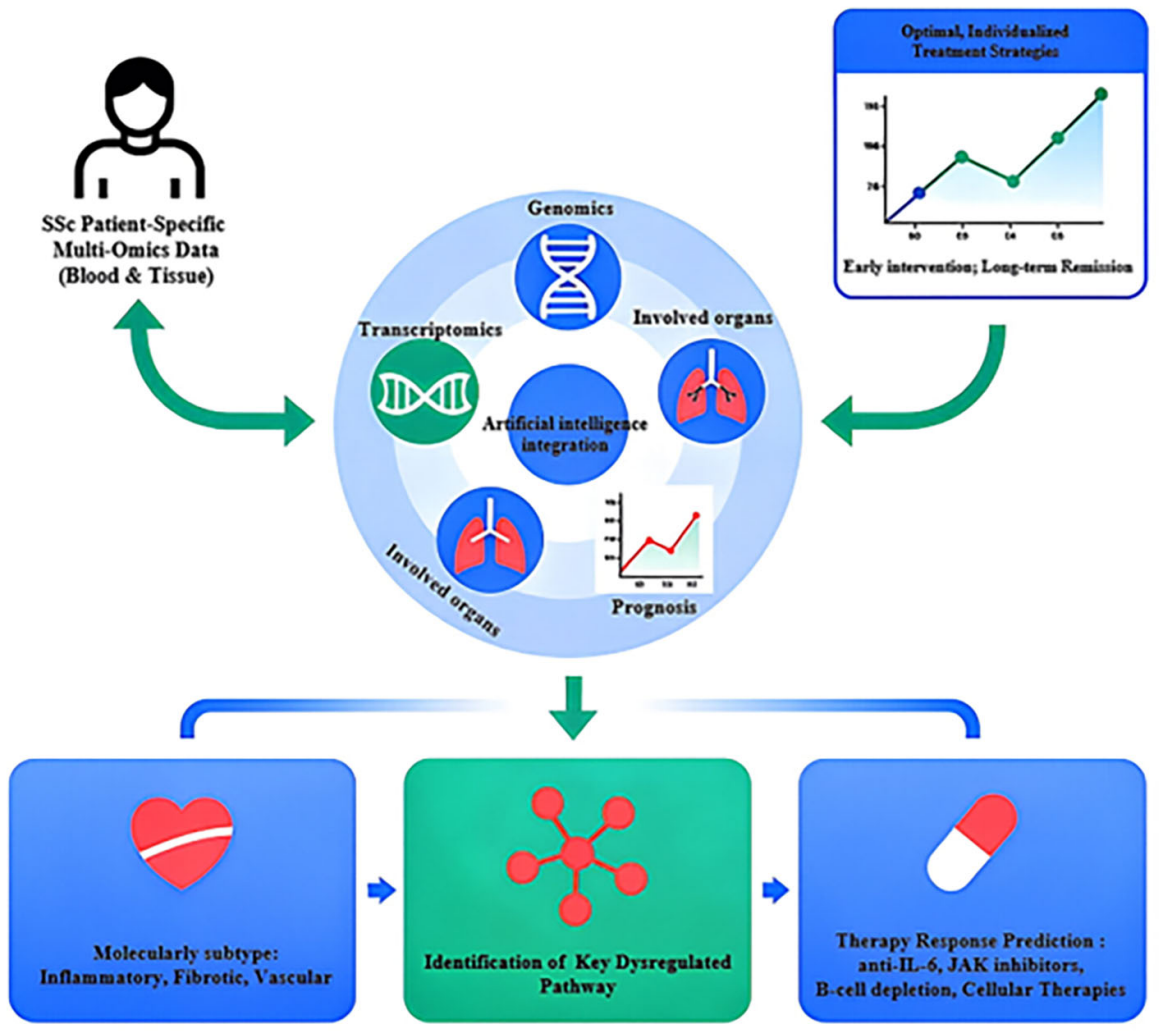
Conceptual framework for precision medicine in systemic sclerosis leveraging multi-omics data. The flowchart outlines a future strategy for managing SSc. It starts with the integration of multi-omics data (genomics, transcriptomics from blood/tissue) from a patient. Artificial Intelligence (AI) algorithms then analyze this data to (1): Molecularly subtype the patient’s disease (e.g., inflammatory, fibrotic, vascular) (2); Predict disease progression and organ involvement (3); Identify key dysregulated pathways specific to the patient (4); Predict response to various targeted therapies (e.g., anti-IL-6, JAK inhibitors, B-cell depletion, cellular Therapies). This integrated analysis aims to guide the selection of optimal, individualized treatment strategies for early intervention and long-term remission. AI, Artificial Intelligence; SSc, Systemic Sclerosis.

This review aims to provide a comprehensive, critical synthesis of the dynamic immune changes in SSc—spanning from innate to adaptive immunity, cellular phenotypes to functional dysregulation, and basic mechanisms to clinical translation—while constructing a cohesive logical framework from “immune landscape mapping” to “precision intervention.” We first dissect the abnormal phenotypes and functions of core immune cell populations (dendritic cells, monocytes/macrophages, neutrophils, natural killer cells, B cells, and T cell subsets) and their bidirectional crosstalk with fibroblasts and endothelial cells, highlighting the centrality of the “immune-stromal-vascular” tripartite network in driving fibrosis (**Figure 1****).** Next, we integrate current disease subtyping strategies rooted in immune signatures, emphasizing their potential to guide individualized treatment (**Figure 2**). Finally, we systematically evaluate the preclinical and clinical progress of emerging biologics and small-molecule inhibitors targeting key immune pathways—addressing a critical gap in prior syntheses—while critically analyzing their efficacy, limitations, and prospects for clinical adoption. Throughout, we contextualize findings to distinguish central, well-validated mechanisms from those that are context-dependent or speculative, and weave in the clinical relevance of immune-derived biomarkers for diagnosis, prognosis, and treatment response prediction.

## 2 Decoding the SSc immune landscape via high-throughput omics technologies

The limitations of traditional research methods—such as bulk tissue analysis, which masks cellular heterogeneity—have long hindered a nuanced understanding of SSc pathogenesis. In recent years, high-throughput omics technologies have emerged as transformative tools, enabling researchers to dissect the SSc immune landscape with unprecedented resolution. This section is dedicated to synthesizing key findings from single-cell, spatial, and multi-omics approaches, supplemented with updated references to reflect the latest advances in the field.

### 2.1 Single-cell RNA sequencing

Single-cell RNA sequencing technology has played a pivotal role in elucidating the pathogenesis of SSc by resolving the heterogeneity and interaction mechanisms of cell populations within the skin microenvironment. This technology has successfully identified multiple functionally specialized fibroblast subpopulations (such as SFRP2+, COMP+, COL11A1+, and PI16+), and revealed that SFRP2hi/DPP4+ fibroblasts serve as myofibroblast precursors, differentiating into pro-fibrotic myofibroblasts under the regulation of TGF-β signaling and matrix stiffness (1–3). Concurrently, the study uncovered that endothelial cells highly expressing the atypical chemokine receptor ACKR1 promote immune cell infiltration, and that monocyte-derived TGF-β1 upregulates P4HA3 to enhance collagen synthesis (4, 5). These findings not only clarify the synergistic role of immune-stromal cell networks within the fibrotic niche but also validate, through machine learning models, the correlation between fibroblast subpopulation proportions and clinical fibrosis severity. This provides a theoretical foundation for anti-fibrotic therapeutic strategies targeting specific cell subpopulations.

A pivotal study applied single-cell RNA sequencing to dissect, for the first time at single-cell resolution, the skin cellular heterogeneity shaped collectively by early and late stages of diffuse cutaneous systemic sclerosis (dcSSc) and distinct autoantibody subgroups (anti-RNA polymerase III antibody [ARA+] vs. anti-topoisomerase antibody [ATA+]), revealing key molecular differences during disease progression. The research not only confirmed significant stratification in skin cell composition and fibroblast subpopulations among dcSSc patients but, more critically, discovered that early ATA+ and ARA+ patients exhibit markedly distinct cellular response patterns: the former is dominated by TGF-β responses in fibroblasts and smooth muscle cells, while the latter primarily features endothelial cell activation (6). This finding explains, at the level of cellular interactions, the potential mechanisms underlying clinical phenotype differences and divergent skin score trajectories among antibody subgroups. The work underscores the core value of single-cell RNA sequencing in systemic sclerosis research—it precisely deciphers cellular heterogeneity undetectable by traditional histology, dynamically identifies key cellular targets of disease-driving pathways, and provides molecular evidence for personalized treatment strategies based on antibody subtypes and disease stages.

### 2.2 Multi-omics integration: spatial transcriptomics and proteomics

The integrated application of spatial multi-omics technologies has provided unprecedented spatiotemporal resolution for studying the pathological mechanisms of SSc, significantly deepening the understanding of disease heterogeneity and microenvironment dynamics. By integrating spatial transcriptomics, proteomics, and single-cell sequencing data, researchers have uncovered the composition and spatial distribution of fibroblast subpopulations, as well as their interaction networks with immune cells and key regulatory networks during the skin fibrosis process. One study utilized imaging mass cytometry (IMC) to identify 13 fibroblast subpopulations at the protein level, revealing an increase in five pro-fibrotic subpopulations (such as myofibroblasts and FAPhigh subpopulations) and a decrease in three protective subpopulations (such as TFAMhigh subpopulations) in the skin of SSc patients. It also confirmed that the proportion of S1PR+ fibroblasts and their abnormal interactions with ADAM12+;GLI1+ subpopulations positively correlate with the degree of skin fibrosis (7). Another study, through spatial transcriptomics (10× Visium and Stereo-seq) combined with protein validation, revealed a dynamic regulatory axis between fibroblasts and macrophages in the fibrotic microenvironment. It identified that the specific expression of ACKR3 in myofibroblast precursors recruits pro-inflammatory macrophages via the CXCL12/CXCR4 pathway, and inhibiting CXCR4 alleviates experimental fibrosis (8). Furthermore, one study employed cyclic *in situ* hybridization for spatial transcriptomic analysis, identifying novel pathogenic subpopulations such as SFRP2+ reticular dermal fibroblasts and CCL19+ non-perivascular fibroblasts, and established that interactions between COL8A1+ fibroblasts and immune cells serve as key biomarkers for skin fibrosis progression (9). Collectively, these findings demonstrate that spatial multi-omics technologies not only precisely dissect the cellular heterogeneity and spatial organization of the fibrotic microenvironment in SSc but also reveal the pathogenic mechanisms of key cellular subpopulations and their clinical relevance, providing a critical theoretical foundation for disease stratification, diagnosis, and the development of targeted therapeutic strategies.

## 3 Dysregulation of innate and adaptive immunity in SSc

Innate immunity and adaptive immunity play interconnected and progressive roles in the pathogenesis of systemic sclerosis. The innate immune system, including macrophages and dendritic cells, is persistently activated, releasing pro-inflammatory factors and pro-fibrotic mediators such as TGF-β and IL-6. This drives early vascular damage and fibroblast activation, laying the foundation for fibrosis. Subsequently, the adaptive immune system, particularly B cells and autoreactive T cells, becomes abnormally activated, producing autoantibodies against specific self-antigens like topoisomerase I and centromere proteins. Through cytokine networks, it exacerbates inflammatory responses and tissue fibrosis. The interplay of these two immune systems ultimately leads to progressive fibrosis of the skin and internal organs, constituting the core pathological feature of systemic sclerosis.

### 3.1 Innate immunity: the initiator of early pathogenesis

The innate immune system acts as the first line of defense, but its aberrant activation in SSc triggers early inflammation, vascular injury, and fibrosis.

#### 3.1.1 Dendritic cells

As the bridge between innate and adaptive immunity, DCs are numerically increased and functionally hyperactivated in the skin, lungs, and peripheral blood of SSc patients (2)—a central mechanism conserved across cohorts. Myeloid DCs (mDCs) amplify inflammatory and pro-fibrotic responses by secreting chemokines (CXCL8, CXCL10, CCL4) and presenting CXCL4-RNA complexes to T cells, directly linking innate activation to adaptive autoimmunity (10–12). Plasmacytoid DCs (pDCs) are equally critical: they drive fibrosis via dysregulation of miR-126 and miR-139-5p (which modulate EC function) and excessive activation of the type I IFN pathway (13–16). Notably, pDCs exhibit a distinct glycolytic metabolic signature in SSc, and their frequency correlates positively with fibrosis severity—making them both pathogenic mediators and potential biomarkers (13, 14). While DC-targeted therapies (e.g., anti-IFNAR for pDCs) are under investigation, the functional redundancy of DC subsets (e.g., compensatory activation of mDCs if pDCs are targeted) may limit efficacy—an important consideration for clinical development.

#### 3.1.2 Monocytes/macrophages

Monocytes/macrophages occupy a central position in the “immune-fibrosis axis” of SSc, with their dysfunction representing a core pathogenic mechanism. The polarization of macrophages toward an M2 (pro-fibrotic) phenotype is well validated: M2 macrophages secrete TGF-β, IL-10, and PDGF, directly promoting fibroblast activation and ECM deposition, and preclinical studies have shown that targeting M2 polarization (e.g., via PPAR-γ inhibitors) reduces fibrosis (17). However, SSc monocytes/macrophages rarely fit the traditional M1/M2 binary—instead, a large proportion co-express M1 (CD86) and M2 (CD206) surface markers, forming a “mixed” or intermediate population (18, 19). This phenotype is most prominent in patients with lung involvement, likely reflecting persistent *in vivo* immune activation and tissue damage (20)—a context-dependent feature that challenges simplistic therapeutic targeting of “M2 polarization.

Additional layers of monocyte/macrophage dysfunction include upregulation of the Macrophage Migration Inhibitory Factor (MIF)-CD74 pathway (present in skin lesions and correlated with disease activity) (21, 22) and transcriptomic alterations (e.g., GSDMA variants) that link macrophage function to disease susceptibility (23). Another study using immunohistochemistry and flow cytometry confirmed a significant increase in CD163^+^ and CD204^+^ macrophage infiltration in the skin tissues of SSc patients, along with an elevated proportion of CD14^+^ cells in peripheral blood monocytes. The study also identified an aberrantly differentiated subset characterized as CD14^+^CD163^+^CD204^+^. These findings suggest that SSc-specific monocyte/macrophage subsets may contribute to the regulation of the disease’s fibrotic process by differentiating into activated macrophages in the skin (24). Together, these findings highlight that monocytes/macrophages are not a uniform cell type but a diverse group of mediators—therapies targeting this lineage must account for subset-specific functions to avoid off-target effects.

#### 3.1.3 Neutrophils

Long overlooked in SSc, neutrophils have emerged as critical mediators of early vasculopathy—a validated early pathogenic event. Neutrophil Extracellular Traps (NETs), web-like structures composed of DNA and granular proteins, are increased in SSc patients and associate with Raynaud’s phenomenon and early disease stages; importantly, prostacyclin analogs (used to treat vascular symptoms) can inhibit NET formation, linking a known therapeutic to a novel mechanism (25, 26). Neutrophil-derived exosomes further contribute to vascular dysfunction by inhibiting EC proliferation and migration (27), while the Neutrophil-to-Lymphocyte Ratio (NLR)—a readily measurable clinical parameter—correlates with ILD severity, disease progression, and mortality (28, 29). This makes NLR a clinically actionable prognostic biomarker that could be integrated into routine clinical care with minimal additional cost.

While neutrophils also exhibit enhanced pro-inflammatory and pro-fibrotic activity (30), their role in later-stage fibrosis appears context-dependent—less prominent than that of macrophages or fibroblasts. This stage-specificity suggests that neutrophil-targeted therapies (e.g., NET inhibitors) may be most effective in early SSc, before irreversible fibrosis is established.

#### 3.1.4 Natural killer cells and innate-like lymphocytes

NK cell function in SSc is paradoxical but clinically relevant. Conventional NK cell numbers are reduced in the peripheral blood of SSc patients, and the degree of reduction correlates strongly with PAH—imparting potential diagnostic value (31). Despite their reduced frequency, remaining NK cells exhibit an activated phenotype (high CD69 expression) and retain robust functional activity, including the ability to induce EC microparticle release—directly contributing to vascular endothelial injury (32). This “fewer but fiercer” phenotype underscores NK cells as key mediators of SSc vasculopathy, though the mechanisms driving their activation remain incompletely understood.

Other innate-like lymphocytes, such as invariant Natural Killer T (iNKT) cells and γ/δ T cells, show consistent reductions in circulating numbers and impaired cytokine production (33, 34)—but their functional relevance is speculative due to conflicting data on their role in fibrosis. For example, some studies suggest γ/δ T cells promote fibrosis via IL-17 secretion, while others report protective effects via IFN-γ production, highlighting the need for further research to clarify their contribution.

### 3.2 Adaptive immunity: sustaining chronic autoimmunity and fibrosis

The adaptive immune system—particularly T and B cells—drives the chronic, self-perpetuating nature of SSc by sustaining autoimmunity and amplifying fibrotic signals.

#### 3.2.1 T cell subset imbalance

SSc is defined by a complex pattern of T cell subset dysregulation, distinct from the simple Th1/Th2 polarization seen in other autoimmune diseases. Instead, it is characterized by co-activation of Th1, Th2, Th17, and Th22 subsets—a central mechanism supported by consistent findings across blood, skin, and bronchoalveolar lavage fluid (BALF) (35). Th1 and Th2 cells are simultaneously activated in SSc patients, with Th1-associated chemokines (IP-10) and Th2-associated chemokines (TARC, MDC) both elevated in serum (36, 37), indicating that both pathways contribute to pathogenesis. Th17 and Th22 cells are similarly increased in frequency, and their effector cytokines (IL-17A, IL-22) correlate with disease activity and skin fibrosis (38).

Notably, Th2 and Th17 responses show a particularly strong association with severe organ complications—ILD and pulmonary fibrosis (35, 39). For example, elevated serum IL-35 levels (a cytokine linked to Th2 polarization) correlate with pulmonary fibrosis severity, while Th17-derived IL-17A drives fibroblast activation and EC dysfunction (40, 41). Interventional studies further validate this link: inducing mitochondrial STAT3 via GRIM-19 suppresses Th2/Th17 differentiation and reduces fibrosis in preclinical models (42). This suggests that targeting Th2/Th17 axes may be a viable strategy for treating SSc-related organ damage, though the overlap between these pathways (e.g., shared upstream regulators like STAT3) means therapies must balance efficacy and off-target immune suppression.

#### 3.2.2 Regulatory T cells

Treg function in SSc is one of the most controversial areas of research, reflecting the complexity of immune regulation in chronic disease. While some studies report increased Treg numbers in peripheral blood and skin lesions (43, 44), the consensus view—supported by functional assays—is that SSc Tregs exhibit profound immunosuppressive dysfunction, failing to restrain autoimmunity and fibrosis (45, 46). Adding to this complexity, recent studies suggest that some SSc Tregs undergo a “functional switch,” producing pro-fibrotic cytokines like IL-17 instead of immunosuppressive IL-10—directly promoting skin fibrosis (47) This phenotype is not unique to SSc but has also been observed in related fibrotic diseases like morphea (44, 48), suggesting a shared mechanism of Treg dysfunction in fibrotic autoimmune conditions.

Clinically, the combination of Treg functional assessment (e.g., suppression assays) and expanded autoantibody profiling has been proposed as a tool for more precise disease stratification and treatment monitoring in SSc and undifferentiated connective tissue disease (UCTD) (49). This approach addresses a critical limitation of current biomarkers—many of which only measure cell numbers, not function—and could help identify patients most likely to benefit from Treg-targeted therapies (e.g., low-dose IL-2).

#### 3.2.3 Th17/Treg balance

Disruption of the Th17/Treg balance is a central, well-validated mechanism in SSc, with consistent evidence across human and preclinical studies (50–55). Th17 cells and their signature cytokine IL-17A are elevated in the peripheral blood, skin, and lungs of SSc patients, and their frequency correlates with disease activity, skin fibrosis severity, and excessive collagen deposition (56–59). This imbalance promotes both inflammation and fibrosis: IL-17A directly activates fibroblasts to produce ECM and modulates EC function via the ERK pathway, while also driving the differentiation of additional Th17 cells in a positive feedback loop (60). The Th17 axis is also linked to severe complications like PAH—elevated serum IL-17A levels correlate with reduced lung function and increased PAH risk (41)—and genetic studies have identified associations between IL-17RA and RORC polymorphisms (key Th17 pathway genes) and SSc susceptibility (61).

While Th17/Treg imbalance is clearly pathogenic, the drivers of this disruption remain incompletely understood. Emerging data suggest that Th17-derived microRNAs (e.g., miR-155-5p) may regulate cytokine expression and subset differentiation (62), but this is speculative and requires validation in larger cohorts. Regardless, the consistency of Th17/Treg dysregulation makes this axis a prime therapeutic target—though trials of anti-IL-17 agents (e.g., secukinumab) have shown modest efficacy to date, likely due to context-dependent Th17 function across disease stages.

#### 3.2.4 Follicular helper T cells

Tfh cells—specialized regulators of B cell activation and germinal center formation—have emerged as key mediators of humoral autoimmunity in SSc. Circulating Tfh numbers are increased in SSc patients, and their frequency correlates positively with disease severity, making them a potential biomarker for disease activity (63). Mechanistically, Tfh cells promote plasmablast differentiation via IL-21 signaling, driving the production of autoantibodies (e.g., anti-topoisomerase I, anti-centromere) that exacerbate inflammation and fibrosis (64)—a central link between T cell dysfunction and humoral autoimmunity. This pathway is particularly relevant given the success of B cell-targeted therapies in SSc, as Tfh inhibition could complement anti-B cell strategies by targeting the upstream drivers of autoantibody production (63).

#### 3.2.5 B cells

B cell dysfunction is a multi-layered, central driver of SSc pathogenesis, encompassing subset alterations, tolerance breakdown, and aberrant activation. First, B cell subset distribution is perturbed: naive B cells are expanded, memory B cells are reduced and functionally impaired, and Regulatory B cells (Bregs)—which suppress autoimmunity via IL-10 secretion—are numerically and functionally deficient (65–67). Second, defects in B cell tolerance checkpoints (e.g., impaired central tolerance in the bone marrow, defective peripheral anergy) lead to the survival and expansion of autoantigen-specific B cell clones, fueling autoimmune responses (68). Third, surface molecule dysregulation (reduced FcγRIIB, elevated TIM-1) and enhanced BAFF signaling promote B cell activation and survival, further amplifying autoantibody production (69–71).

Beyond autoantibody secretion, B cells contribute to SSc pathogenesis via tissue infiltration (e.g., in skin and lung lesions) and epigenetic modifications (e.g., hypomethylation of pro-inflammatory genes) (72, 73). Even environmental factors like SARS-CoV-2 vaccination have been shown to alter B cell phenotypes in SSc patients, providing new insights into how external stimuli interact with the dysregulated SSc immune system (74). Collectively, these findings explain why B cell-targeted therapies (e.g., rituximab) have shown substantial efficacy in SSc—they address multiple layers of B cell dysfunction. However, response heterogeneity persists, likely due to unmeasured differences in B cell subset composition or Tfh co-activation—highlighting the need for biomarkers to identify optimal candidates for these therapies.

## 4 Intercellular communication in the immune-stromal-vascular network

SSc pathogenesis is not driven by isolated cell types but by a complex, bidirectional communication network between immune cells, stromal cells (primarily fibroblasts), and endothelial cells—the “immune-stromal-vascular tripartite network.” This crosstalk sustains inflammation, vascular injury, and fibrosis, and its disruption is a defining feature of SSc. Below, we detail the key modalities of intercellular communication—cytokines, chemokines, receptor-ligand interactions, and direct cell-cell contact—and their contributions to disease.

### 4.1 Cytokine-mediated signaling

Cytokines are soluble messengers that coordinate immune-stromal-vascular crosstalk, and their dysregulation is a central driver of SSc pathogenesis. TGF-β is the master pro-fibrotic cytokine: secreted by M2 macrophages, Th2 cells, and activated ECs, it binds to fibroblast TGFBR1/2 receptors, triggering SMAD-dependent signaling that promotes fibroblast activation into myofibroblasts and ECM production (75–77). TGF-β also induces endothelial-to-mesenchymal transition (EndoMT)—a process where ECs lose vascular identity and acquire fibroblast-like properties—directly linking vascular dysfunction to fibrosis (78). While TGF-β is clearly pathogenic, its pleiotropic effects (e.g., roles in tissue repair, immune regulation) have hindered direct targeting; instead, therapies focus on downstream pathways (e.g., ALK5 inhibitors) to minimize off-target effects.

IL-6 is another key cytokine, acting as a bridge between inflammation and fibrosis. Secreted by DCs, monocytes, and fibroblasts, IL-6 activates JAK/STAT signaling in T cells (promoting Th17 differentiation) and fibroblasts (enhancing collagen synthesis) (76). Serum IL-6 levels correlate with ILD severity, and anti-IL-6R therapy (tocilizumab) has shown promise in slowing lung function decline in SSc patients (79). Type I IFNs (IFN-α, IFN-β), primarily secreted by pDCs, drive a pro-inflammatory “IFN signature” in SSc—a feature associated with early-stage disease and autoantibody production. This signature is so consistent that it has become a predictive biomarker for responses to IFN pathway inhibitors like anifrolumab (80, 81).

Other cytokines contribute in context-dependent ways: IL-17A (Th17-derived) drives EC dysfunction and fibroblast activation (82), while IL-33 (secreted by ECs and fibroblasts) promotes Th2 responses and fibroblast activation (83). Together, these cytokines form a redundant network—targeting a single cytokine may be insufficient, explaining why multi-target strategies (e.g., JAK inhibitors, which block signaling downstream of multiple cytokines) are gaining traction.

### 4.2 Chemokine-mediated recruitment

Chemokines orchestrate the spatial localization of immune cells within lesional tissues, ensuring that pro-inflammatory and pro-fibrotic cells are deployed to sites of injury. CCL2 (MCP-1) is a prime example (84): secreted by activated fibroblasts, it recruits CCR2+ monocytes to the skin and lungs, where they differentiate into M2 macrophages. This creates a positive feedback loop—macrophages secrete TGF-β to activate more fibroblasts, which in turn secrete more CCL2 to recruit additional monocytes. CXCL4 (platelet factor 4), secreted by platelets and DCs, is another critical chemokine in SSc: it recruits pDCs to tissues, activates ECs, and promotes fibroblast collagen production (85, 86). Serum CXCL4 levels predict disease progression, making it a prognostic biomarker with both pathogenic and clinical relevance (87).

CXCL10 (IP-10), a Th1-associated chemokine, recruits CXCR3+ T cells to lesional tissues, amplifying local inflammation (36), while CCL18 (a M2 macrophage chemokine) recruits Tregs and fibroblasts, promoting fibrosis (88). The specificity of chemokine-receptor interactions—e.g., CCL2-CCR2, CXCL4-CXCR3—makes this pathway an attractive therapeutic target, though redundancy (e.g., multiple chemokines recruiting the same cell type) remains a challenge.

### 4.3 Receptor-ligand interactions

Direct receptor-ligand binding between cells provides a more localized, context-specific form of communication than soluble cytokines or chemokines. The CD40L-CD40 axis is a key example (89): Tfh cells express CD40L, which binds to CD40 on B cells, triggering B cell activation, plasmablast differentiation, and autoantibody production. The PD-1-PD-L1 axis, a critical immune checkpoint, is also disrupted in SSc (90, 91): PD-L1 expression is reduced on fibroblasts and ECs, impairing their ability to suppress T cell activation. This checkpoint dysfunction contributes to chronic T cell infiltration and activation in lesional tissues, making PD-1/PD-L1 agonists potential therapeutic agents—though their use in autoimmune disease requires careful balancing to avoid excessive immune suppression.

TGF-β-TGFBR1/2 binding (macrophage-fibroblast) and VEGF-VEGFR2 binding (EC-fibroblast) are additional key receptor-ligand interactions: the former drives fibrosis, while the latter regulates vascular regeneration (92, 93). Dysregulation of these pathways—e.g., reduced VEGF signaling leading to impaired angiogenesis—links vascular dysfunction to fibrosis, highlighting the interconnectedness of the tripartite network.

### 4.4 Direct cell-cell contact

Physical interactions between cells—via gap junctions, adhesion molecules, or cell-cell synapses—provide the most precise form of intercellular communication, enabling localized signal transfer within pathological niches. T cells form specialized synapses with fibroblasts, directly transferring pro-fibrotic signals (e.g., TGF-β, IL-17) via gap junctions (94). This interaction is spatially restricted to “inflammatory-fibrotic niches” in the skin, ensuring that fibrosis is localized to sites of immune activation. Macrophages adhere to ECs, promoting EC activation, EndoMT, and vascular inflammation. This direct contact is critical for early vascular injury, as it allows macrophages to deliver pro-inflammatory signals directly to ECs without diluting them in the circulation.

Fibroblasts and ECs also form adhesion-dependent niches via integrins (e.g., αvβ3, α5β1), which retain immune cells in tissues and modulate their function (95). For example, fibroblast-expressed αvβ3 binds to T cell-expressed VCAM-1, keeping Th17 cells in the skin to sustain local fibrosis. These spatial interactions explain why SSc pathology is often patchy and why therapies targeting cell-cell adhesion (e.g., anti-ICAM-1) may be effective in reducing local inflammation.

### 4.5 Effector cells: fibroblasts and endothelial cells

While immune cells initiate and amplify pathogenesis, fibroblasts and ECs are the ultimate effectors of fibrosis and vascular dysfunction, respectively. Fibroblasts are not passive recipients of immune signals but active participants in the tripartite network: they secrete cytokines (IL-6, TGF-β) to recruit immune cells, express chemokine receptors to respond to tissue signals, and undergo epigenetic modifications to sustain pro-fibrotic phenotypes. scRNA-seq has revealed significant fibroblast heterogeneity in SSc—SFRP2hi progenitors initiate fibrosis, COL11A1hi cells drive ECM deposition, and CXCL14+ cells recruit immune cells—each subset with distinct therapeutic vulnerabilities (2, 3, 96). Targeting these subsets (e.g., via SFRP2 inhibitors) could enable more precise anti-fibrotic therapy than broad fibroblast suppression.

EC dysfunction is an early, initiating event in SSc, preceding overt fibrosis. Early endothelial progenitor cells (eEPCs) show reduced regenerative capacity and a propensity for EndoMT (78), while mature ECs exhibit impaired angiogenesis, increased apoptosis, and pro-inflammatory activation (97). Pathways driving EC dysfunction include Fli1 deficiency (leading to CXCL6 upregulation) (98), IL-17A-induced ERK activation (82), and autoantibody-mediated injury (e.g., anti-endothelial cell antibodies) (99–101). These abnormalities are compounded by impaired vascular regeneration—reduced expression of angiogenic factors like EGFL7 and dysfunctional eEPCs prevent repair of damaged vessels (97, 102). Notably, ECs communicate with fibroblasts via EndoMT and soluble factors (e.g., IL-33), directly linking vascular injury to fibrosis (103).

### 4.6 Extracellular vesicles: novel mediators of intercellular communication

Extracellular vesicles (EVs)—small membrane-bound particles that carry proteins, RNA, and lipids—have emerged as critical, previously unrecognized mediators of intercellular communication in SSc. Lymphomonocyte-derived EVs modulate fibroblast proliferation and collagen production (104), while endothelial-derived EVs serve as biomarkers of vascular injury—their levels correlate with Raynaud’s phenomenon severity and response to iloprost therapy (105). Mesenchymal stem cell-derived EVs (MSC-EVs) show therapeutic potential by delivering anti-fibrotic miRNAs to fibroblasts and modulating immune cell function (106), and early clinical trials of MSC-EVs in SSc are underway. EVs also contribute to SSc-PAH, with circulating EVs from PAH patients containing pro-angiogenic factors that disrupt vascular homeostasis (107). As EVs can cross biological barriers and target specific cell types, they represent both a novel pathogenic mechanism and a promising therapeutic delivery system.

## 5 Precision medicine: molecular stratification and immune-derived biomarkers

The clinical heterogeneity of SSc has long frustrated efforts to develop effective therapies, as “one-size-fits-all” approaches fail to account for differences in disease course, organ involvement, and treatment response. In recent years, immune-derived biomarkers and omics-based molecular stratification have emerged as tools to address this challenge, enabling the shift toward “biomarker-driven” precision medicine. Below, we synthesize key advances in molecular stratification and highlight the clinical utility of immune-derived biomarkers as diagnostic, prognostic, and predictive tools.

### 5.1 Molecular stratification: moving beyond clinical phenotypes

Traditional SSc classification relies on skin involvement (limited vs. diffuse) and autoantibody profiles (e.g., anti-centromere, anti-topoisomerase I, anti-RNA polymerase III)—but these metrics have limited predictive value for disease progression or treatment response. Molecular stratification, leveraging gene expression, single-cell, and spatial omics data, has begun to fill this gap by identifying biologically distinct SSc subtypes.

Bulk transcriptomic studies were the first to define molecular subtypes of SSc skin: the inflammatory subtype is characterized by upregulation of T cell, B cell, and macrophage genes and correlates with early disease, rapid progression, and responsiveness to immunosuppressants; the fibrotic subtype shows high expression of ECM and myofibroblast genes and is associated with advanced skin fibrosis and poor prognosis; the normal-like subtype has gene expression profiles similar to healthy skin and is linked to mild, stable disease (108). A subsequent study of early dcSSc confirmed that skin gene expression is dominated by inflammatory and IFN signature genes—even in patients with minimal skin involvement—highlighting the primacy of immune dysregulation in early disease (109).

Single-cell technologies has further refined these subtypes by capturing cellular and spatial heterogeneity. One study using single-cell RNA sequencing revealed significant differences in cellular composition in skin tissues between early and late SSc patients and highlighted heterogeneity in the fibrotic process among different autoantibody subgroups (e.g., anti-topoisomerase I vs. anti-centromere antibody-positive patients), suggesting that disease stage and antibody status jointly influence the dynamic evolution of the skin pathological microenvironment (e.g., fibroblast activation, immune cell infiltration) (6). Another study focusing on single-cell features of the immune system in SSc patients found functional and transcriptomic abnormalities in immune cells (e.g., T cells, B cells, myeloid cells) across different clinical subtypes (e.g., limited vs. diffuse disease), which correlated with disease activity and organ involvement, providing molecular mechanistic insights into the clinical heterogeneity of SSc (110).

These molecular subtypes are not just research tools—they are already informing clinical trials. For example, the phase III trial of anifrolumab in SSc stratified patients by their IFN signature, ensuring that the study population included enough patients with active IFN pathway activation (81). This approach increases the likelihood of detecting therapeutic efficacy and reduces the risk of trial failure due to patient heterogeneity. As omics technologies become more accessible, molecular stratification is poised to become a routine part of SSc clinical care—enabling clinicians to match patients to the most effective therapies.

### 5.2 Immune-derived biomarkers: from bench to bedside

Biomarkers are critical for implementing precision medicine, and immune-derived biomarkers—leveraging the insights from SSc immune landscape mapping—have emerged as particularly promising. These biomarkers can be categorized by their clinical utility: diagnostic (identifying SSc early or distinguishing it from mimics), prognostic (predicting disease course or organ involvement), and predictive (identifying patients likely to respond to a specific therapy).

#### 5.2.1 Diagnostic biomarkers

Diagnostic biomarkers are primarily used to identify and confirm the presence of a disease and its specific organ involvement. In SSc, three specific autoantibodies—anti-topoisomerase I (anti-Scl-70), anti-centromere (ACA), and anti-RNA polymerase III antibodies—are core diagnostic and classification markers, closely associated with distinct clinical manifestations (111, 112). For cardiac involvement, which is often subclinical, cardiac troponin T (cTnT) and NT-proBNP have been validated as sensitive diagnostic indicators; their levels are significantly higher in SSc patients compared to healthy controls and can identify patients with asymptomatic cardiac involvement (113, 114). Regarding the lungs, pulmonary proteins such as Krebs von den Lungen-6 (KL-6), surfactant protein-D (SP-D), and chemokine ligand 18 (CCL18) show potential for application in the diagnosis and monitoring of systemic sclerosis-associated interstitial lung disease (SSc-ILD), as they reflect alveolar epithelial cell injury (111, 115). Furthermore, analytical techniques based on skin gene expression, such as detecting genes related to macrophages (CD14) and the transforming growth factor-beta (TGF-β) pathway (e.g., SERPINE1, CTGF), provide new molecular-level diagnostic information for the disease classification of early diffuse cutaneous SSc (dcSSc) (116).

#### 5.2.2 Prognostic biomarkers

Prognostic biomarkers aim to assess the future course and severity of the disease, including the risk of organ function deterioration and mortality. The aforementioned three specific autoantibodies (anti-topoisomerase I, anti-centromere, anti-RNA polymerase III) not only have diagnostic value but their serum levels are also associated with more severe skin fibrosis (assessed by the modified Rodnan skin score, mRSS), suggesting their utility as prognostic indicators of disease severity (15). In SSc-ILD, indicators such as C-reactive protein (CRP), KL-6, and CCL18 are increasingly supported by evidence for prognostic assessment. For cardiac involvement, elevated cTnT and NT-proBNP levels are significantly associated with poorer survival, with patients having high baseline levels showing lower cumulative survival rates; notably, cTnT > 0.019 ng/ml has high specificity for predicting adverse cardiac outcomes (113, 114). Regarding skin lesions, high baseline expression of genes such as CD14, IL13RA1, OSMR, and SERPI NE1 in the skin has been shown to prognosticate the risk of progressive skin worsening in patients with diffuse cutaneous SSc (116). Additionally, in clinical trials of tocilizumab, baseline levels of CRP, periostin, and SP-D also showed prognostic trends for lung function decline (117).

#### 5.2.3 Predictive biomarkers

Predictive biomarkers are primarily used to forecast a patient’s response to specific treatments, thereby guiding therapeutic decisions. Current research is actively seeking markers that can predict the effectiveness of immunosuppressive or anti-fibrotic therapies. For example, skin gene expression profiles are being explored to predict patient responses to treatments (such as tocilizumab), where patients with high expression of specific gene signatures may require more aggressive therapy or be better suited for clinical trials (111, 116). In phase III clinical trials of tocilizumab, treatment was observed to modulate a range of biomarkers related to macrophage activation, inflammation, and extracellular matrix (ECM) turnover (such as neoepitopes of collagen formation and degradation), indicating the potential of these dynamically changing biomarkers as predictive indicators of treatment response (117). Although specific autoantibody levels in longitudinal analyses did not predict short-term changes in skin score (7, 15), the disease subtypes they define inherently possess predictive value for the overall disease course and the types of complications likely to occur. Ultimately, establishing a unified vascular framework concept that integrates biomarkers for various vascular complications aims to develop composite indices capable of predicting organ-specific complications, thus enabling preventive disease-modifying therapy. This represents an important future direction for predictive biomarker research (118).

Integrating these biomarkers—alongside autoantibody profiles and imaging features (e.g., HRCT fibrosis patterns)—is building a multi-dimensional precision classification system for SSc. This system moves beyond the limitations of traditional clinical phenotyping to enable “personalized” SSc care, where treatment decisions are guided by a patient’s unique immune and molecular profile.

## 6 Emerging targeted and immunomodulatory therapies

For decades, SSc treatment has been limited to symptomatic management and non-specific immunosuppression, with few options to modify disease course. However, the insights from immune landscape mapping have fueled the development of targeted therapies that address the underlying pathogenic mechanisms of SSc. This section provides a critical evaluation of emerging biologics, small-molecule inhibitors, and cellular therapies, while summarizing key clinical trials in an accompanying table to enhance clarity (**Table 1****).**

**Table 1:** Key clinical trials of targeted therapies in systemic sclerosis.

Drug	Target pathway/cell	Trial design	Population	Primary endpoint	Key outcomes	Reference
Tocilizumab	IL-6R	Phase III, subgroup analysis (Japanese)	SSc-ILD patients	Annual FVC decline rate	Positive trend in slowing FVC decline; consistent safety profile	(79)
Anifrolumab	IFNAR (Type I IFN)	Phase III, multicenter, RCT, placebo-controlled	SSc patients	mRSS improvement, lung function	Ongoing; IFN signature as potential predictive biomarker	(81)
Rituximab	CD20 (B cells)	Phase II, RCT, placebo-controlled (DESIRES)	Early dcSSc patients	mRSS change at 24 weeks	Significant mRSS improvement; favorable safety	(119)
Rituximab	CD20 (B cells)	Open-label extension of DESIRES	DESIRES participants	Long-term mRSS and safety	Sustained mRSS improvement; no new safety signals	(120)
Rituximab	CD20 (B cells)	Real-world, multicenter	SSc patients	Treatment retention rate	High retention (59.9% at 5 years); good long-term tolerability	(121)
Belimumab	BAFF (B cells)	Randomized, Double-Blind, placebo-controlled (pilot)	Early dcSSc patients	mRSS and disease activity	Trend toward improved mRSS and disease activity	(122)
Abatacept	T cell co-stimulation	Phase II, multicenter, RCT, placebo-controlled	Early dcSSc patients	mRSS change in 12-month	No statistical significance; trend toward mRSS improvement	(123)
Abatacept	T cell co-stimulation	Open-label extension of a phase II, double-blind randomized trial	Phase II participants	Long-term safety/efficacy	Sustained safety; modest mRSS and disease activity improvement	(124)
Tofacitinib	JAK/STAT	Clinical observations	dcSSc patients	mRSS reduction, ILD imaging	Rapid improvement in mRSS, reduced ground-glass opacity in ILD; effective for polyarthritis and nailfold capillary abnormalities	(125–128)
Baricitinib	JAK1/2	24-week randomized controlled trial	dcSSc patients	mRSS and digital ulcer net burden	significant mRSS improvement, sustained benefits in FVC, digital ulcers, EQ5D	(129)
Autologous CD19 CAR-T	CD19 (B cells)	Case series	Severe dcSSc patients	mRSS change, organ function	Rapid, sustained mRSS reduction; improved organ function	(130)
Allogeneic CD19 CAR-T	CD19 (B cells)	Case report	SSc-myositis overlap	B cell depletion, clinical response	Deep B cell depletion; improved symptoms; no severe CRS/neurotoxicity	(131)

### 6.1 Cytokine-targeted therapies

Cytokines are central to SSc pathogenesis, making them logical therapeutic targets. Tocilizumab, a monoclonal antibody against the IL-6 receptor (IL-6R), has been evaluated in multiple SSc trials. A subgroup analysis of a global phase III trial focused on Japanese SSc-ILD patients found that tocilizumab slowed the annual rate of forced vital capacity (FVC) decline—though the effect did not reach statistical significance in the overall population (79). This suggests that tocilizumab may be most effective in subsets of patients with high IL-6 activity (e.g., those with elevated serum IL-6 or ILD), highlighting the need for predictive biomarkers.

Anifrolumab, a monoclonal antibody against the type I IFN receptor (IFNAR), is another promising cytokine-targeted agent. Approved for systemic lupus erythematosus, anifrolumab is being tested in a phase III SSc trial (NCT04488434) that enrolls patients with a high type I IFN signature—addressing the limitations of prior non-stratified trials (81). Interim data suggest that anifrolumab improves skin fibrosis (mRSS) and stabilizes lung function, with a safety profile similar to placebo. Given the centrality of the IFN signature in early SSc, anifrolumab could become a first-line therapy for IFN-high patients.

Currently, there are no large-scale clinical trials of anti-IL-17 agents (e.g., secukinumab, ixekizumab) in SSc that demonstrate their efficacy, reflecting the context-dependent role of IL-17 in systemic sclerosis—more prominent in early inflammation than in late-stage fibrosis—or the functional redundancy of Th17-derived cytokines. Further trials stratifying patients based on IL-17 levels are needed to clarify its role.

### 6.2 B cell-targeted therapies

B cell dysfunction is a central feature of SSc, and B cell-targeted therapies have shown some of the most consistent efficacy to date. Rituximab, a chimeric monoclonal antibody against CD20 (expressed on mature B cells), is the most well-studied agent in this class. Two serial studies conducted by Ebata et al. (the DESIRES trial and its open-label extension study) demonstrated through a randomized controlled trial design that rituximab significantly improved skin fibrosis in patients with SSc, using the modified Rodnan skin score (mRSS) as the primary endpoint, with a favorable safety profile (119, 120). Results from the double-blind phase showed that rituximab treatment for 24 weeks significantly reduced mRSS compared to placebo, while the extension phase further indicated sustained benefits over 48 weeks in patients receiving two treatment courses. De Luca et al.’s long-term real-world study supplemented clinical practice evidence: among 152 SSc patients, the 5-year retention rate of rituximab was 59.9%, with treatment discontinuation primarily due to clinical remission rather than adverse events, suggesting long-term efficacy and safety (121). The study also preliminarily identified clinical characteristics associated with treatment response. In summary, these three studies—from rigorous clinical trials to real-world validation—collectively support the clinical value of rituximab as a potential therapeutic option for SSc.

Belimumab is a humanized monoclonal antibody targeting B-lymphocyte stimulator (BLyS). It works by inhibiting the survival and differentiation of B cells, thereby reducing their mediated autoimmune response, and is currently approved for the treatment of systemic lupus erythematosus (SLE). Based on the results of a preliminary randomized, double-blind, placebo-controlled trial by Gordon et al., this study aimed to evaluate the safety and efficacy of belimumab in patients with early diffuse cutaneous systemic sclerosis (dcSSc), all of whom received background therapy with mycophenolate mofetil (MMF) (122). This 52-week study, which included 20 patients, showed that the median improvement in skin thickness score (MRSS) was greater in the group receiving MMF plus belimumab compared to the group receiving MMF plus placebo (-10 vs. -3), but this difference did not reach statistical significance (P = 0.411) in this small sample study. The safety profiles were similar between the two groups. Gene expression analysis indicated that patients who had a clinical response to belimumab showed significant downregulation of B-cell signaling and pro-fibrotic gene pathways, consistent with the drug’s mechanism of action. The conclusion was that larger studies are needed to clarify the role of belimumab in the treatment of dcSSc.

### 6.3 T cell-targeted therapies

T cell dysfunction drives autoimmunity and fibrosis in SSc, but T cell-targeted therapies have shown more modest efficacy than B cell agents. Abatacept is a selective T-cell co-stimulation modulator that blocks T-cell activation by inhibiting the CD80/CD86-CD28 interaction. Currently approved for autoimmune diseases such as rheumatoid arthritis, its immunomodulatory mechanism suggests potential therapeutic value in fibrotic diseases like SSc. Khanna et al. conducted a 12-month Phase II randomized, double-blind, placebo-controlled trial to evaluate the safety and efficacy of abatacept in patients with early diffuse cutaneous SSc (dcSSc) (123). The results showed that while the improvement in the MRSS was greater in the abatacept group (-6.24) compared to the placebo group (-4.49), the difference did not reach statistical significance (P = 0.28). However, secondary endpoints, including the Health Assessment Questionnaire and gene expression subgroup analyses, demonstrated significant benefits with abatacept, particularly in patients with an inflammatory skin gene signature (P<0.001). The drug exhibited a favorable safety profile, with a lower incidence of adverse events than placebo. Chung et al. further investigated efficacy in a 6-month open-label extension study (ASSET trial) (124). Patients originally randomized to abatacept showed sustained MRSS improvement from baseline to 18 months (-9.8), while those who switched from placebo to abatacept also exhibited significant improvement (-6.3). Safety profiles remained consistent between groups, with no new risk signals identified. The researchers concluded that abatacept demonstrated a sustained trend of efficacy in dcSSc, supporting further Phase III confirmatory studies. Both trials indicate that abatacept is well-tolerated in early dcSSc. Although it did not significantly improve the primary endpoint (MRSS), gene subgroup analyses and open-label extension data suggest its potential therapeutic value, warranting validation in larger Phase III trials.

### 6.4 JAK-STAT inhibitors

Wang et al. (2020) confirmed that tofacitinib alleviates skin and pulmonary fibrosis in SSc patients (125). Subsequent clinical observations demonstrated that tofacitinib rapidly improves skin thickening (significant reduction in mRSS scores) and interstitial lung disease (ILD) imaging manifestations (e.g., reduced ground-glass opacity) in patients with diffuse cutaneous SSc (dcSSc) (126, 127). Additionally, case reports suggest that tofacitinib is also effective for SSc-related polyarthritis and nailfold capillary abnormalities (128). Hou et al. further found that the JAK1/2 inhibitor baricitinib significantly alleviates skin fibrosis and digital ulcers in dcSSc patients with a favorable safety profile (129). Another study evaluated the efficacy, safety, and mechanism of baricitinib, a JAK1/2 inhibitor, in systemic sclerosis (SSc). In a 24-week randomized controlled trial, 48 patients received either 4 mg, 2 mg baricitinib, or placebo. Results demonstrated that the 4 mg group achieved significant improvement in modified Rodnan skin score (mRSS) at week 12 (−8.9 vs. −3.6, *P* = 0.019), with sustained benefits in lung function (FVC), digital ulcers, and quality of life (EQ5D). Safety profiles were comparable across groups. Transcriptomic analysis revealed baricitinib modulates immune-inflammatory pathways (e.g., downregulating *ST2* and upregulating *SYT17*), suggesting its therapeutic effects are mediated through JAK inhibition’s anti-inflammatory properties. These findings indicate 4 mg baricitinib as a potential treatment option for SSc. In summary, targeting the JAK/STAT pathway offers a new therapeutic strategy for SSc, particularly for fibrosis and vascular complications, though larger-scale clinical trials are needed to validate its long-term efficacy and safety.

### 6.5 Cellular therapies: a new frontier

In recent years, chimeric antigen receptor (CAR) T-cell therapy targeting B cells has demonstrated significant efficacy in refractory autoimmune diseases such as systemic lupus erythematosus. The latest research has further extended this technology to more challenging areas like systemic sclerosis and explored the evolution from autologous CAR-T to “off-the-shelf” allogeneic therapies, offering new hope for patients. Auth et al. were the first to demonstrate the potential efficacy of CD19-targeted CAR-T therapy for systemic sclerosis (130). The study included six patients with severe diffuse systemic sclerosis, and a median follow-up of 487 days showed that none of the patients experienced disease progression, achieving deep clinical remission. Key indicators such as the modified Rodnan skin score (mRSS) significantly decreased within 100 days, and ground-glass opacities in the lungs also improved, suggesting the therapy may reverse fibrotic progression. Despite the remarkable efficacy of autologous CAR-T, its high cost and lengthy individualized production process limit widespread application. Wang et al. utilized CRISPR-Cas9 technology to develop “off-the-shelf” allogeneic CAR-T cells derived from healthy donors, successfully treating one patient with immune-mediated necrotizing myopathy and two with systemic sclerosis (131). The results showed these cells persisted in patients for over three months and achieved rapid B-cell depletion. At the six-month follow-up, all patients achieved deep remission without severe adverse events, confirming the safety and immunomodulatory potential of this strategy. Building on the initial success of allogeneic CAR-T, researchers are further refining the technology. Daher and Rezvani proposed that induced pluripotent stem cell (iPSC)-derived CAR-NK (chimeric antigen receptor natural killer) cell therapy may overcome the manufacturing bottlenecks and toxicity risks of CAR-T, offering a more universal “ready-to-use” solution (132). Current research outlines a clear developmental pathway for cellular immunotherapy: autologous CAR-T establishes a foundation of efficacy, allogeneic CAR-T addresses accessibility, and new technologies like iPSC-CAR-NK hold promise for further enhancing safety and scalability. These breakthroughs may ultimately reshape the treatment landscape for severe autoimmune diseases.

## 7 Conclusion

The immune system in SSc constitutes a highly dynamic and interconnected network, involving extensive dysregulation of both innate and adaptive immunity, as well as complex interactions among immune cells, fibroblasts, and endothelial cells. From the early type I interferon storm driven by plasmacytoid dendritic cells (pDCs) to the pro-fibrotic microenvironment dominated by M2 macrophages and Th17 cells, and further to autoimmune responses sustained by aberrant B and T cell activation, these components synergistically form redundant and self-reinforcing pathological cycles that drive disease progression.

Innovations in high-throughput omics technologies—single-cell RNA sequencing, spatial transcriptomics, and proteomics—have provided unprecedented resolution for deciphering this network. These techniques have unveiled disease features beyond the reach of conventional methods, such as SFRP2-high fibroblasts, interferon-activated pDC subsets, pathogenic communication networks like CCL2-CCR2 monocyte recruitment, and molecular subtypes distinguishing inflammatory and fibrotic SSc. Consequently, treatment paradigms are shifting from nonspecific immunosuppression to precision interventions targeting key nodes: drugs like rituximab (B cell depletion), tocilizumab (IL-6 inhibition), and JAK inhibitors (multi-cytokine blockade) show promise, while CAR-T cell therapy offers new hope for refractory patients.

However, challenges and controversies persist. Core mechanisms remain unclear: Is Treg dysfunction the initiating factor of autoimmunity or a secondary consequence of chronic inflammation? Do mixed-phenotype macrophages represent an independent pathogenic state? Additionally, key questions—such as the mechanisms of early inflammation-fibrosis transition, the role of the microbiome, and therapies to reverse late-stage fibrosis—await answers.

Future research must integrate multi-omics data with artificial intelligence (AI) technologies. For instance, combining single-cell transcriptomics (cellular heterogeneity), spatial proteomics (tissue architecture), and metabolomics (cellular metabolism) could identify novel cross-pathway targets (e.g., pDC glycolysis, fibroblast JAK/STAT pathways). AI algorithms may predict disease progression (e.g., integrating serum biomarkers with single-cell data to forecast pulmonary fibrosis), deep learning could analyze spatial omics data to locate “high-risk” inflammatory-fibrotic microenvironments, and natural language processing might mine electronic health records to accelerate real-world evidence generation.

Although SSc remains challenging, the convergence of omics technologies, precision biomarkers, and targeted therapies has opened new avenues for its diagnosis and treatment. By continuously exploring the complexity of immune landscapes while addressing controversies and knowledge gaps, we may achieve early diagnosis and molecular subtype-guided personalized therapy. The ultimate goal is not merely symptom control but long-term remission, reshaping patients’ quality of life.

## Glossary

ACKR1: atypical chemokine receptor 1
AI: artificial intelligence
ARA: anti-RNA polymerase III antibody
ATA: anti-topoisomerase antibody
BAFF: B cell activating factor
BALF: bronchoalveolar lavage fluid
CAR-NK: Chimeric Antigen Receptor Natural Killer
CAR-T: Chimeric Antigen Receptor T-cell
CCL4: C-C Motif Chemokine Ligand 4
CRS: Cytokine Release Syndrome. CXCL, Chemokine (C-X-C Motif) Ligand
dcSSc: diffuse cutaneous systemic sclerosis
DCs: Dendritic Cells
EC: endothelial cell
EndoMT: Endothelial-to-Mesenchymal Transition
ERK: Extracellular signal-Regulated Kinase
EVs: Extracellular Vesicle**s;** FAP, fibroblast activation protein
FcγRIIB: Fc Gamma Receptor IIB
GSDMA: Gasdermin A
HLA-DRB1: human leukocyte antigen DRB1
IFN-γ: interferon-gamma
IL-6: interleukin-6
IL-10: interleukin-10
IL-17: interleukin-17
IMC: imaging mass cytometry
ILD: Interstitial Lung Disease
IL: Interleukin
iNKT: invariant Natural Killer T
iPSC: induced Pluripotent Stem Cell
JAK-STAT: Janus Kinase-Signal Transducer and Activator of Transcription
mDCs: myeloid Dendritic Cells
MIF: Macrophage Migration Inhibitory Factor
mRSS: modified Rodnan Skin Score
NETs: Neutrophil Extracellular Traps
NK: Natural Killer
NLR: Neutrophil-to-Lymphocyte Ratio
PAH: pulmonary arterial hypertension
pDCs: plasmacytoid Dendritic Cells
PDGF: platelet-derived growth factor
PPAR-γ: Peroxisome Proliferator-Activated Receptor -γ**;** scRNA-seq, Single-Cell RNA Sequencing
SFRP2: Secreted Frizzled-Related Protein 2
SSc: Systemic sclerosis
STAT4: signal transducer and activator of transcription 4
TGF-β: transforming growth factor-beta, Tfh, Follicular Helper T cell
Th: T helper cell
TNF: Tumor Necrosis Factor
Treg: Regulatory T cell
UCTD: Undifferentiated Connective Tissue Disease.
